# The unequal face of hunger: how gender and race/colour have exacerbated food insecurity during the COVID-19 pandemic—a cross-sectional analysis

**DOI:** 10.1186/s12889-025-24427-z

**Published:** 2025-09-24

**Authors:** Gleiciane Bueno da Silva Luiz, Aline Alves Ferreira, Rosana Salles-Costa

**Affiliations:** 1 Postgraduate Program in Nutrition, Josué de Castro Nutrition Institute, Federal University of Rio de Janeiro, Avenida Carlos Chagas Filho, 373, Bloco J, 2° andar, sala 18 – Cidade Universitária, Rio de Janeiro, RJ 21941-902 Brazil; 2 Department of Social and Applied Nutrition, Josué de Castro Nutrition Institute, Federal University of Rio de Janeiro, Rio de Janeiro, RJ Brazil

**Keywords:** Gender inequality, Racism, Food insecurity, COVID-19

## Abstract

**Background:**

The COVID-19 pandemic has aggravated Food Insecurity (FI) in a heterogeneous way across different household profiles. The aim of this study was to assess the influence of the first two years of the COVID-19 pandemic on FI in Brazil according to the intersections of race/colour and the gender of household heads.

**Methods:**

Microdata from the 1st and 2nd Food Insecurity Surveys in the Context of the COVID-19 Pandemic (VIGISAN) were used (1st VIGISAN: n = 2,180; 2nd VIGISAN: n = 12,745). FI levels were compared according to four groups for the head of households (White men, Black men, White women and Black women). Odds ratio (OR) values were calculated using multinomial logistic regression models to assess the association between reference person profiles and FI levels. Marginal effects were estimated after adjusting the final model.

**Results:**

In Brazilian households, the proportion of Food Security (FS) decreased, while levels of Food Insecurity (FI) increased. The chances of being in moderate + severe FI increased significantly, especially in households headed by Brown/Black women, from 2.2 (95% CI 1.3;3.7) to 3.2 (95% CI 2.5;4.0).

**Conclusions:**

The results of this study reinforce the need to plan equitable public policies that debate the intersectionality of gender and race/colour as a way of guaranteeing food and nutritional security in Brazil.

## Background

Racial and gender inequality is a persistent problem in many countries, such as Brazil. The interaction between these inequalities promotes disparities in access to essential services for human development, such as food and nutrition. During the COVID-19 pandemic, these vulnerabilities increased. It is therefore necessary to understand how these structural inequalities affect FI in the face of a new viral pandemic. We searched the PubMed, Google Scholar, LILACS, and CAPES databases for information on the prevalence of FI in Black women both before and during the COVID-19 pandemic. We used search terms in both English and Portuguese to find publications dated between 2018 and 2024. The search terms included “food insecurity”, “sexism”, “racism” and “COVID-19”. Studies that investigate FI considering the intersectionality of gender and race/skin colour in the context of the COVID-19 pandemic remain scarce. For data analysis, we used data from a nationally representative survey of the Brazilian population called the National Survey on Food Insecurity in the Context of the COVID-19 Pandemic (VIGISAN). During the pandemic, this was the only survey that provided data on food security (FS) and FI levels nationwide.

Research focused on FI considering gender and race/skin colour during the pandemic remains scarce. This study provides important data and reflections for better understanding, based on intersectionality, how gender and race/colour have interacted dynamically during the COVID-19 pandemic, particularly reflecting the situation of FS/FI. In addition, the findings of the study support black movements that are fighting for anti-racist and anti-patriarchal public food and nutrition policies.

During the COVID-19 pandemic, being part of the Black and female population has been a major factor in the levels of FI in Brazilian homes, revealing the persistence of prejudice and the lack of government assistance aimed at this population group. The findings shed light on this group that is constantly made invisible despite its active participation in various spheres of society. Finally, we emphasize the importance of considering race/skin colour and gender intersectionality in the analysis of FI in the context of COVID-19 to provide support for the formulation of public policies that meet the demands of this population, focusing mainly on reducing the most serious levels of FI.

## Introduction

In 2020, the world was surprised by a new global public health crisis caused by a highly transmissible and lethal virus that profoundly altered all spheres of humanity [1]. However, in the socioeconomic and demographic spheres, the consequences have been more pronounced. The measures necessary to contain the spread of the virus, such as lockdowns, have led to the loss of jobs or sources of income [2]. As a result, economic instability has made it difficult for many families to access resources to face the pandemic without jeopardising their well-being and access to good food and nutrition. According to the United Nations (ONU), 892.7 million people experienced severe FI between 2020 and 2022 [3]. In Latin America and the Caribbean alone, the number of people living with hunger reached 43.2 million [3].

FI occurs when people do not have secure access to sufficient quantities of safe and nutritious food for normal growth and development and an active and healthy life [4]. In addition, the concept of FI used in Brazil emphasises that it can have significant impacts on health, both in terms of social exclusion, loss of self-esteem and emotional stress, as well as compromised nutritional status itself [5]. In other words, FI refers not only to food shortages but also to wider ramifications of such shortages, including social, psychological, and nutritional aspects, which negatively affect people’s quality of life.

Brazil is internationally recognized for its success in tackling hunger. The country was removed from the hunger map by the Food and Agriculture Organization (FAO) in 2014 [6]. A significant part of this success was achieved through the development and implementation of the Brazilian Food Insecurity Scale (EBIA). This indicator is responsible for estimating FI at the household level [5]. However, since 2016, political and economic conditions have become unfavourable and incompatible with the agenda of promoting food and nutrition security (FNS) [7]. The adoption of austerity policies throughout the country has once again exposed Brazil to high levels of household FI. Thus, with the arrival of the COVID-19 pandemic, concerns about future food supplies, shortages and even hunger became more severe [8, 9]. However, these consequences have been heterogeneously observed in society [2].

Historically, the Black population has suffered from constant violations of basic human and social rights. During the pandemic, it has become clear that the structural effects of racial and gender discrimination have placed this population in even more disadvantaged situations, thereby exacerbating existing social disparities [2, 10]. Thus, analyses that take race/colour and gender into account show that these social indicators relate in different ways to the experience of families dealing with FI [11]. For Black women, the situation is even more evident since the history of racism and sexism in Brazil means that these individuals suffer an overlap of these axes of oppression [12] and are saddled with a greater degree of vulnerability in guaranteeing FS, as evidenced by them suffering from the most severe levels of FI [13, 14].

Thus, it has been observed that while the novel coronavirus disease could potentially affect everyone, the ability to obtain resources to overcome or prevent such infection, as well as manage the related consequences, was not equitable in Brazil. Given that many of Black women serve as heads of household, all the paths led to an unequal increase in household FI in the country during the pandemic [8, 9]. Given this scenario, this study aimed to assess the influence of the COVID-19 pandemic (2020–2022) on FS and FI levels in Brazil according to the race/colour and gender of the head of household.

## Methods

This study was based on microdata from 2 nationally representative surveys assessing FI in the Brazilian population during the COVID-19 pandemic (1st and 2nd VIGISAN) [8, 9] carried out by the Brazilian Food and Nutrition Sovereignty and Security Research Network (Rede PENSSAN) in December 2020 (1st VIGISAN) and November 2021 to April 2022 (2nd VIGISAN). In relation to losses and refusals to each survey, the I VIGISAN [8] 100% of the planned sample was fulfilled. For the II VIGISAN [9], 98.04% of the sample were obtained (less than 2% loss in the sample of 13,000 households estimated for FI estimates in the country.

Both surveys have similar study designs, with a sample base that is representative of the national territory, considering data from Brazilian Census from 2010 to select the sample size. The households were selected from the same census tracts used in the master sample of the Brazilian Institute of Geography and Statistics (IBGE) population surveys. For the selection of households, conglomerate sampling was used in three stages of selection (municipalities, census tracts, households). The 1st VIGISAN obtained a probabilistic sample of 2180 households, with an estimated 95% confidence interval and a maximum margin of error of 2.1 percentage points for the estimates. In the 2nd VIGISAN, 12,745 households were sampled with a maximum margin of error for the total sample of 0.9 percentage points. More details on the sampling design can be found in the methodological section of the surveys. In both surveys, the head of the household was considered the individual responsible for decision-making in the household [8, 9].

The outcome variables analysed were FS and FI levels, which were evaluated by the short version of the Brazilian Household Food Insecurity Measurement Scale (EBIA). The EBIA is a psychometric scale that assesses the interviewee’s perception of access to food, in terms of quality and quantity, in the three months prior to the interview [15]. In the surveys, the validated short version of the EBIA was used to estimate FS and FI levels [16]. The choice to use the 8-item version of the EBIA was due to the need for a rapid population survey to reduce the risk of interviewer contamination. The 8 questions on the scale are dichotomous (yes/no) [16], and each affirmative answer adds one point to the classification of the household’s FI level. If all the questions are negative, the household is classified as FS.

Based on the classifications, the households were classified into either FS or three levels of FI (mild, moderate, and severe). FS classified households considered to have regular and permanent access to high-quality food in sufficient quantity without compromising access to other essential needs. Mild FI represents concern or uncertainty about access to food in the future. Moderate FI refers to quantitative food reduction among adults and/or disruption in eating patterns resulting from food shortages among adults. Severe FI are likely to run out of food, and members of such households are likely to go a day or more without eating during the reference time frame. These criteria are fully consistent with the hunger construct [17]. In this study, the moderate FI and severe FI categories were grouped together as a way of analysing the most severe categories in terms of access to adequate food [13].

To understand the relationship between power systems (gender and race/skin colour) and FI during the pandemic, the intersectionality debate was adopted because its theoretical foundation seeks to capture the structural and dynamic consequences of the interaction between two or more axes of subordination [18]. To do this, the household heads were stratified according to sex and race/skin colour, following methodologies previously used by other studies [13, 14].

Gender was assessed using the biological variable sex, adopted from Brazilian population studies [19]. This variable takes the binary form “male” or “female”. However, for the intersectional debate, we used the gender debate to understand the complexities and nuances that extend beyond biological categories, considering how behaviours between men and women shape individual and collective experiences are related as one of the explanatory arguments, while at the same time structuring inequality. Thus, the variable “gender” will be used to lead the discussions.

Regarding race/skin colour, the self-declaration used in Brazilian population studies was applied [19], which considers five options: White, Black, Yellow, Brown or Indigenous. Given that the 1st and 2nd VIGISAN address samples that do not account for ethnic and racial minorities, it was not possible to represent the indigenous and yellow categories; thus, both were excluded from the analysis (1st VIGISAN: 4.5%; 2nd VIGISAN: 3.4%). In Brazil, the Black population is composed of both Black and Brown individuals because both categories are of African descent and face similar challenges due to the racism rooted in Brazilian society. Therefore, in this study, the categories “Black” and “Brown” were consolidated into a single classification, resulting in the race/skin colour variable having two categories, namely, “White” and “Black/Brown”.

Socio-demographic variables related to households and the head of the household, which are associated with FI, were assessed for each of the surveys. The covariates were selected based on a theoretical review and a systematic review. The variables include: household information: region (North, Northeast, South/Southeast, or Central-West), area of household (urban or rural), number of residents (1–2, 3–5, or > 5), Presence of children under 5 (yes or no) and per capita family income (up to 1 MW or more than 1 MW)—considering the values in force during the reference period of each year: USD 287.79 (BRL 1039.00) in 2020 and USD 327.00 (BRL 1212.00) in 2022. Head of household information: age group, schooling (years of study) (0–8 and > 8), and occupation (family farmer or rural producer, informal worker, formal worker, regular self-employed worker or individual entrepreneur, regular self-employed worker or individual entrepreneur or other (homemaker, pensioner, student, etc.)

For the descriptive analyses, the proportion values and their respective 95% confidence intervals (95% CIs) were estimated according to the same variables for each survey. To compare significant differences between each survey, the 95%CI levels were compared, considering the overlapping of the intervals as an indication of not significant comparison between two prevalence. FS prevalence and levels of FI were calculated according to the profit of the household head. A chi-square test was used to assess the associations between the household head and FS/FI.

In the next stage, separate models (crude and adjusted) were constructed for each survey and the odds ratio (OR) values were calculated using multinomial logistic regression models to assess the association between the profiles of head of household who considered gender and race/skin colour (White man—reference category); White woman; Black man; Black woman), with the outcome levels of FI (using FS as a reference category). The models considered the adjustment for potential confounding variables (schooling, per capita family income, region, and area of the household) in the relationship with FI levels, which were determined based on a systematic review, tested separately (crude bivariate multinomial models) and included in the final adjusted model for those that obtained a p value < 0.05 (data not shown).

The marginal effects were estimated for the FI outcome according to the profiles of the head of household after adjusting the final model [20], separately for each survey (I VIGISAN and II VIGISAN). The values of the variation in the probability of the outcome (FS/FI) were plotted according to the profiles of the head of the household (Fig. 2).

The statistical analysis was carried out considering the complex sample design, 95% CI, using the ‘svy’ commands of Stata software, version 16.1 (StataCorp LLC, College Station, 2016), for this purpose.

The utilised survey is part of a broad project aiming to monitor FS/FI in the context of COVID-19, coordinated by the PENSSAN Network and carried out by the Vox Populi Institute. This study was approved by the Research Ethics Committee of the Clementino Fraga Filho University Hospital of the Federal University of Rio de Janeiro—CAEE 30679914.0.0000.5257. This study uses databases whose information is aggregated without the possibility of individual identification; thus, it was not necessary to submit this information to the ethics committee of the National Research Ethics Commission (CONEP) according to Resolution No. 510 of April 7, 2016.

## Results

In Brazil, the proportion of households headed by Black/Brown individuals, regardless of gender, was greater than that of households headed by other categories (Table 1). In addition, comparing the two surveys, there was an increase in the proportion of heads of household with informal occupations and those who had regular jobs or who were individual entrepreneurs in the second year of the survey. The proportions of FS and mild FI decreased in 2022 (FS = 41.3%; mild FI = 28.0%) in comparison to 2021 (FS = 44.8%; mild FI = 34.7%), while the more severe forms of FI (moderate + severe) increased (2021 = 20.5%; 2022 = 30.7%). The variations in FI levels were found to be significant when comparing the 95% CIs (Table 1).

**Table 1:** Description of the sociodemographic characteristics of reference persons and households in Brazil, 2020–2022

Characteristics	BRAZIL			
	I VIGISAN (2021)		II VIGISAN (2022)	
	%^a^	95% CI^b^	%^a^	95% CI^b^
Characteristics of the head of household				
*Race/colour and gender profile*^c^				
White man	20.4	18.1–22.9	19.1	18.0–20.2
White woman	17.9	15.7–20.3	17.4	16.4–18.5
Black/Brown man	31.4	28.9–34.1	32.2	31.0–33.4
Black/Brown woman	30.3	27.8–32.9	31.3	30.1–32.6
*Age (years)*				
Up to 24	5.0	4.0–6.3	5.5	4.91–6.03
25–39	22.8	20.5–25.1	26.5^e^	25.4–27.7
40–59	43.0	40.3–45.9	42.3	41.0–43.7
60 or more	29.2	26.7–31.8	25.7	24.5–26.9
*Schooling (years of study)*				
0–8	49.7	49.9–52.5	47.1	45.8–48.5
> 8	50.3	47.5–53.1	52.9	51.6–54.2
*Occupation*				
Family farmer or rural producer	6.2^e^	5.2–7.4	3.1	2.7–3.4
Informal worker	14.9	13.0–17.0	17.8	16.9–18.8
Formal worker	21.0	18.8–23.4	25.2	24.0–26.4
Regular self-employed worker or individual entrepreneur	14.7	12.8–16.9	16.3	15.3–17.4
Unemployed	7.8	6.4–9.5	7.9	7.2–8.7
Other (homemaker, pensioner, student, etc.)	35.4	32.6–38.0	29.7	28.6–31.0
Household characteristics				
*Area*				
Urban	85.6	84.1–87.1	85.5	84.7–86.2
Rural	14.4	12.9–15.9	14.5	13.8–15.3
*Region*				
Central-West	7.7	7.0–8.5	7.6	7.2–10.0
North east	26.2	24.1–28.4	26.0	25.1–27.0
North	7.5	6.8–8.2	6.9	6.6–7.3
Southeast/South	58.6	56.1–61.1	59.5	58.3–60.6
*Number of residents*				
1–2	40.6	37.9–43.3	50.7	49.4–52.0
3–5	53.1^e^	50.3–55.9	45.4	44.4–46.7
> 5	6.3	5.2–7.7	3.9	3.5–4.5
*Presence of children under 5*				
Yes	13.2	11.5–15.2	15.1	14.2–16.1
No	86.8	84.8–88.5	84.9	83.9–85.8
*Per capita family income (Minimum wage*^d^*)*				
Up to ½ MW	43.3	40.4–46.2	30.6^e^	33.4–36.2
More than 1 MW	56.7	53.8–59.6	69.4	68.1–70.7
*Food security and Levels of Food insecurity (FI)*				
Security	44.8	42.0–47.6	41.3	40.0–42.7
Mild Insecurity	34.7	32.1–37.4	28.0	26.8–29.2
Moderate + Sereve Insecurity	20.5	18.4–22.7	30.7	29.4–32.0

^a^Proportions (%), ^b^Confidence intervals (95% CI), ^c^Black men and women were considered to be the combination of Black and Brown race/colour; ^d^Considering the values in force during the reference period of each year: USD 287.79 (BRL 1039.00) in 2020 and USD 327.00 (BRL 1212.00) in 2022. ^e^*p* value < 0.05

**Fig. 1:**
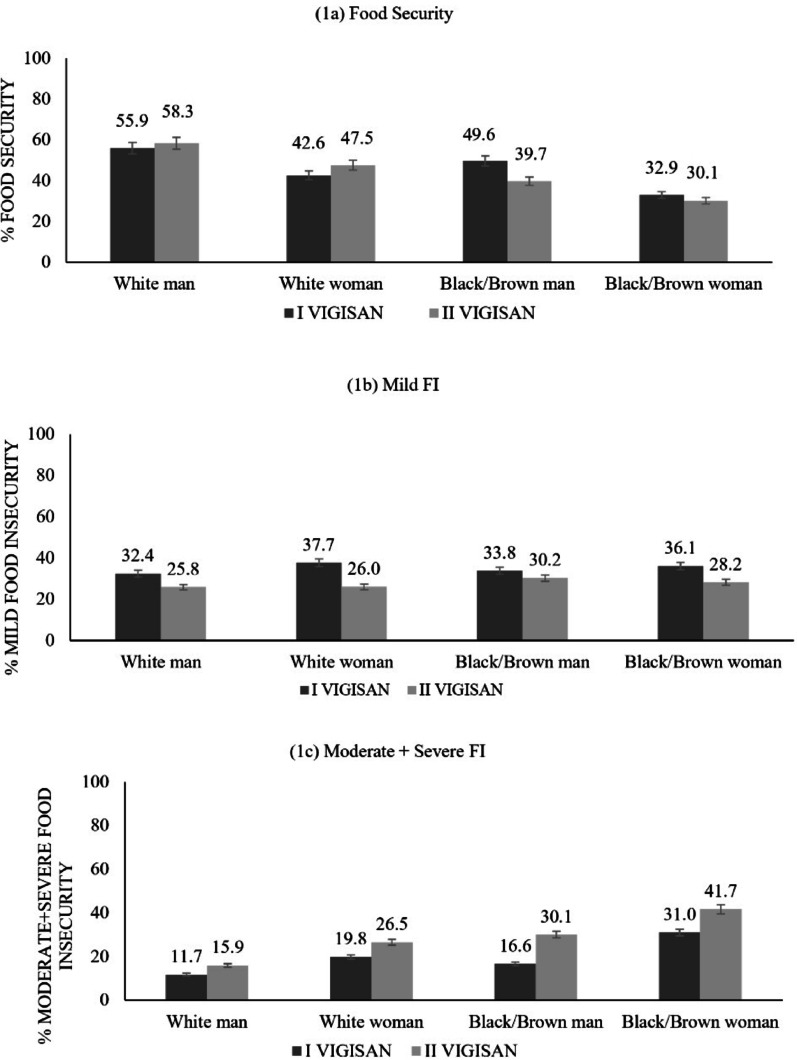
Prevalence (%) of food insecurity according to race/colour and sex in the households in Brazil, 2020–2022. *Note* Black men and women are the combination of Black and Brown race/colour

When analyzing the scenario of change in FS and FI levels according to race/colour and gender profiles (Fig. 1), it was observed that households headed by White people showed an increase in FS (*p* value < 0.05), while in households headed by Black/Brown people the prevalence was significantly lower. With regard to severe FI levels, regardless of the fact that the increase was significant in both White and Black headed households, it is worth noting that when the Black woman was in this position, the increase was significantly greater.

According to the ORs in the final adjusted model (Table 2), in 2020, the chance of moderate + severe FI was significantly greater among households headed by women, mostly when a Black/Brown woman was in this position (OR = 2.2; 95% CI 1.3; 3.7). After one year of the COVID-19 pandemic, the chance of mild FI was greater among households headed by both Black/Brown men and women (*p* value < 0.001). However, after one year of COVID-19 pandemia, the chance of mild FI was significantly higher among households headed by a Black/Brown (*p* value < 0.001). Regarding the chance of moderate + severe FI, although it was higher among all profiles (*p* value < 0.001), it was among families headed by a Black/Brown woman that the risk was greatest. In this group of families, the OR was 3.2, with a variation of up to 4.0 when considering the upper limit of the 95% CI on the adjusted modelling (Table 2).

**Table 2:** Odds ratios and 95% confidence intervals of sociodemographic variables and food insecurity by gender and race/colour in Brazil, 2020–2022

Gender and race/colour profile	I VIGISAN (2020)						II VIGISAN (2022)					
	Crude model			Adjusted model^b^			Crude model			Adjusted model^b^		
	OR	95% CI	*p value*^c^	OR	95% CI	*p value*^c^	OR	95% CI	*p value*^c^	OR	95% CI	*p value*^c^
*Mild FI*												
White man	1.0^a^											
White woman	1.5	1.0–2.4	0.058	1.5	0.93–2.45	0.091	1.2	1.0–1.6	0.08	1.2	0.9–1.5	0.25
Black/Brown man	1.2	0.8–1.7	0.395	1.0	0.64–1.54	0.995	1.7	1.4–2.1	< 0.001	1.4	1.1–1.7	< 0.001
Black/Brown woman	1.9	1.3–2.9	< 0.001	1.4	0.88–2.13	0.156	2.1	1.7–2.6	< 0.001	1.5	1.2–1.9	< 0.001
*Moderate* + *Severe FI*												
White man	1.0^a^											
White woman	2.2	1.3–3.9	< 0.001	1.8	1.0–3.3	0.05	2.0	1.6–2.6	< 0,001	1.9	1.5–2.6	< 0.001
Black/Brown man	1.6	1.0–2.6	0.05	1.1	0.6–1.8	0.82	2.8	2.3–3.4	< 0,001	1.9	1.5–2.4	< 0.001
Black/Brown woman	4.5	2.8–7.2	< 0.001	2.2	1.3–3.7	< 0.001	5.1	4.3–6.2	< 0,001	3.2	2.5–4.0	< 0.001

^a^Model reference category, Food Security; ^b^Models adjusted for household location (urban/rural), household region (North, Northeast, South/Southeast, Midwest), schooling in years of study (Up to 8 years/9 years or more) and per capita household income (Up to 1 MW/More than 1 MW)—considering the values in force during the reference period of each year: USD 287.79 (BRL 1,039.00) in 2020 and USD 327.00 (BRL 1,212.00) in 2022; ^c^Consider p-value significant when <0.05.

**Fig. 2:**
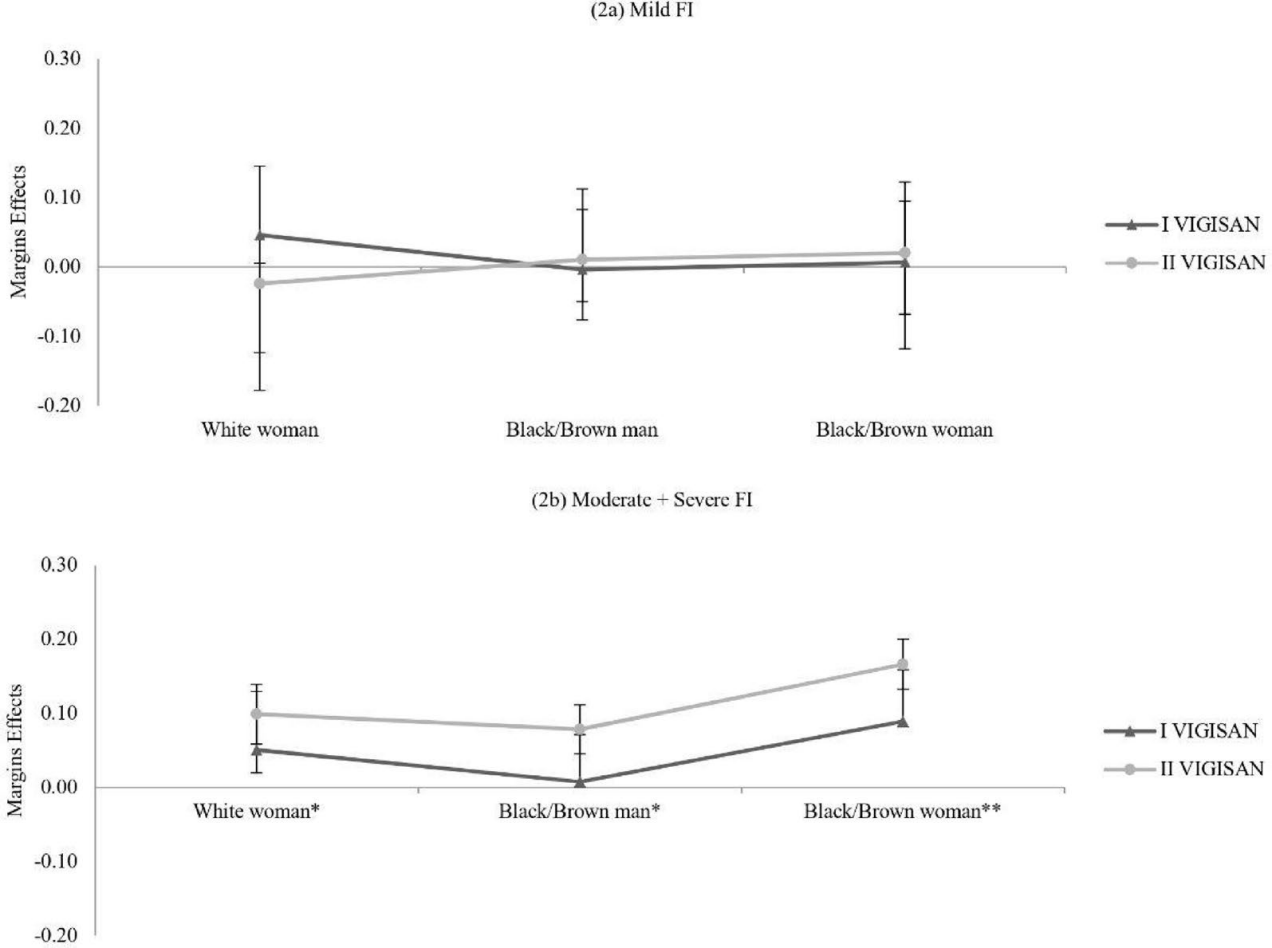
Distribution of marginal effects for food insecurity according to sex and race/colour in Brazil (2020–2022). *Notes* Marginal effects estimated by multinomial logistic regression models for each survey. *P value < 0.05 in II VIGISAN. **P value < 0. 05 in both surveys

This vulnerability is evidenced by the marginal effects shown in Fig. 2, where the variation in the probability of moderate/severe FI was significantly higher in households headed by Black women in both surveys. In the period between the two surveys, the variability value of the probability of a family headed by a Black woman increased from 0.09 to 0.17 (*p* value < 0.001). Households headed by White women also showed a significant increase, from 0.05 to 0.10 (*p* value =  < 0.001), in the variation in the probability of living with moderate/severe FI. Among Black men, the probability of moderate/severe FI increased from 0.01 to 0.08, becoming statistically significant only in the second year of the pandemic (*p* value =  < 0.001).

## Discussion

In the first years of the pandemic, families headed by Black people had a worse FS status, to the detriment of an increase in all levels of FI. However, when a Black woman was the head of the household, families were up to 4 times more likely to have moderate/severe FI.

The significant increase in FI in families where the reference variable was female or Black/Brown in the first two years of the pandemic can be explained by the decrease in family income. Normally, the same social characteristics are described for Black/Brown women, i.e., working in undervalued professions, having a high probability of living in homes with a high number of residents, unfavourable conditions and precarious economic conditions, and performing multiple tasks (productive and reproductive life) [2, 21–23]. The paralysis of activities such as education, cultural events and nonessential businesses during the pandemic affected various economic activities that depended on the high level of movement of people in urban centres, as well as work in labour spaces [24]. As a result, the likelihood of layoffs and wage cuts, with a consequent increase in the number of unemployed and informal jobs, has tended to be greater among Black and low-income women [2].

These results must be analysed from the point of view of the combination of racism and sexism, which in turn can produce a form of “social asphyxiation”, which is a term used by Sueli Carneiro that has negative repercussions on all dimensions of life [12]. This statement shows that the experience of being a Black/Brown woman in Brazil is accompanied by the possibility of experiencing the worst living conditions. As several possible factors explain this scenario, it is common for the lower-level Brazilian families to have female members working in informal or lower-paying jobs, with less schooling, and living in unhealthy housing and basic sanitation conditions [22].

Because these axes of oppression are structural conditions, negative consequences, such as hunger, were already observed before the pandemic began. In the study by Santos et al*.* [13], families headed by single Black women with at least one child younger than 5 years old were three times more likely to live with moderate/severe FI in both 2004 and 2013. However, this likelihood became four times greater in 2018, when FI began to increase again in Brazil. In another study carried out in the city of Salvador (northeast of the country), where 80% of the population is self-declared Black and Brown, the prevalence of FI was found to be greater in households headed by Black women (mild FI: 25.6% and moderate or severe FI: 21.2%) [14].

The findings of these previous studies help us understand that the scenario of experiencing FI was already being conditioned mainly among families headed by Black/Brown women. Although scientific evidence shows the vulnerabilities of this population group, there are few instances of public policies that consider the intersectionality of gender and race/colour in their formulations. Thus, it is clear that this situation is not just a result of the COVID-19 pandemic. In fact, the health crisis has exposed social weaknesses and increased vulnerabilities as a result of socioeconomic damage.

To alleviate the socioeconomic crisis, in 2020, the federal government adopted a policy of income transfer by distributing so-called emergency aid as a social protection measure [25]. This was a financial benefit created with the aim of guaranteeing a minimum income for Brazilians during the COVID-19 pandemic. It was aimed at informal workers, individual microentrepreneurs, the self-employed and/or the unemployed [25]. However, recent studies have shown that this program has not been sufficient to curb the increase in severe FI in Brazil [7]. Delays in decision-making, bureaucratic procedures for accessing the benefits implemented and difficulties in using digital technology are some of the issues that may have contributed to this situation among vulnerable populations [26]. In addition, the decrease in the value of this emergency aid at the beginning of 2021 and the failure to grant it, especially during the period when the 2nd VIGISAN data collection began, may have contributed to the significant increase in the most severe forms of FI.

Adding to the debate on these results is the fact that household chores and caring for children and adolescents overlap in families. According to Rodrigues et al. [23], the priority given to women in the reproductive sphere also contributes to their being less involved in productive work in Brazilian families, especially when this responsibility establishes a class relationship. The authors also point out that for women living in poverty, reconciling both productive and reproductive work intensifies the overload resulting from social responsibilities. The availability of public facilities to reduce the overload of tasks considered feminine, such as community kitchens, school meals, crèches and full-time schools, is important.

In addition, in the first two years of the COVID-19 pandemic, in addition to being unpaid, female heads of household had their feminine duties overloaded in their homes due to the closure of nurseries and schools and the increase in the number of people infected and sick with the virus; this may also have resulted in an increase in FI in female-headed households [8, 9].

The compulsory closure of school networks has forced children and adolescents to remain at home. Globally, the lives of millions of people in this group have been impacted by this measure, requiring emergency adaptation to the remote-homeschooling model [27]. As a result, there has been an increase in the social responsibilities of female heads of household, coupled with the intense mental burden that the pandemic scenario has placed on them.

Furthermore, in Brazil, according to the 2023 School Census [28], the majority of children enrolled in early childhood education are Black/Brown. Many of these children benefit from the Brazilian National School Feeding Program (PNAE), which is a national program that covers students from the entire Brazilian public basic education network (early childhood education, elementary education, high school and youth and adult education) enrolled in public schools [29]. Among the PNAE’s guidelines is the formation of healthy eating habits through the provision of meals and food and nutrition education. At the time of the school closures in Brazil, the federal government authorized the distribution of food purchased with PNAE funds to the parents or guardians of students in public basic education schools at the discretion of the local government during the period when classes were suspended [29]. However, importantly, these measures may not have been enough to contain the spread of FI, as the transfer of such aid did not guarantee that the person responsible had access to food in terms of quantity and quality, and the amount of food transferred to schools has not been adjusted since February 2017, despite the increase in food prices [30] compromising the quality of the meals served [31].

Women are still primarily responsible for caring for the home, children, elderly people and sick people. Although much of the unpaid care work in the world, especially in low-income countries, was already performed by women before the COVID-19 pandemic, there has been a significant increase in this burden. The resulting negative impact on women is likely to last for years [10].

Regarding the possible limitations of this study, even though the data analysed are representative of the Brazilian population, the debate on gender issues is limited since the “sex” variable adopted in the VIGISAN surveys was used; this variable is based on IBGE population surveys and was collected with only two response options (male and female), thereby excluding other representations of gender identities. Furthermore, I VIGISAN has a smaller sample size than II VIGISAN, which may be a limitation when comparing the data. However, both used the 2010 Census as their sampling base and followed the same selection methodology, which reduces methodological bias.

Another important point to consider in relation to the possibility of this study being limited is the fact that the proportion of Black and Brown people is higher than that of the White population. In this sense, it is important to note that the results described in this manuscript follow the national trend of FI prevalence in the Black population, as observed in a study published by Santos et al. [13]. In that study, the authors evaluated a national sample representative of the Brazilian population before the covid-19 pandemic, and the relationship between race/colour was similar to that described in this study.

The findings in this article highlight that the problems caused by structural inequalities and the COVID-19 pandemic still represent a challenge for reducing inequalities in access to healthy food for the Brazilian population. However, it is important to add to the debate on the increase in severe forms of FI in Brazil that public policies aimed either directly or indirectly at promoting food and nutritional security in Brazil began to be dismantled in 2016, which contributed to this setback in the fight against hunger [7]. Although there are policies aimed at the Black population and those aimed at women, the two policy streams are not in dialogue. This situation poses a greater risk in times of crisis. These are important factors that may have contributed to the increase in inequalities marked by the debate on the intersectionality of gender and race discussed in this article.

At the beginning of 2023, Brazil resumed its fight against hunger as one of the goals of the current federal government. The National Council for Food and Nutrition Security (CONSEA), which was dissolved at the beginning of 2019, was reestablished to include monitoring, planning and evaluating food and nutrition security policies. Recently, the 6th National Conference on Food and Nutritional Security recognized, in its first thematic axis, the need for state participation in overcoming inequalities, especially those related to racism and patriarchy, among other structural determinants [32].

Therefore, the results of this article corroborate the need to structure actions and strategies and reformulate public policies aimed at ensuring the eradication of hunger and guaranteeing the human right to adequate food through anti-racist and anti-patriarchal food systems.

## Data Availability

The data that support the findings of this study are available from PENSSAN Network but restrictions apply to the availability of these data, which were used under license for the current study, and so are not publicly available. However, data may be available by contacting the PENSSAN Network (https://pesquisassan.net.br/).

## Abbreviations

CAPES: Coordination of superior level staff improvement
CONEP: Committee of the national research ethics commission
CONSEA: Council for food and nutrition security
EBIA: Brazilian household food insecurity measurement scale
FAO: Food and Agriculture Organization
FI: Food insecurity
FNS: Food and nutrition security
FS: Food security
IBGE: Brazilian Institute of Geography and Statistics
ONU: United Nations
OR: Odds ratio
PENSSAN Network: Food and nutrition sovereignty and security research network
PNAE: Brazilian national school feeding program
VIGISAN: Insecurity surveys in the context of the COVID-19 pandemic
